# Teriparatide Improves Bone and Lipid Metabolism in a Male Rat Model of Type 2 Diabetes Mellitus

**DOI:** 10.1210/en.2019-00239

**Published:** 2019-07-23

**Authors:** Sachiko Nomura, Akihiro Kitami, Ryoko Takao-Kawabata, Aya Takakura, Momoko Nakatsugawa, Ryohei Kono, Akihiro Maeno, Akihiko Tokuda, Yukihiro Isogai, Toshinori Ishizuya, Hirotoshi Utsunomiya, Misa Nakamura

**Affiliations:** 1 Department of Strategic Surveillance for Functional Food and Comprehensive Traditional Medicine, Wakayama Medical University, Wakayama, Japan; 2 Clinical Development Center, Asahi Kasei Pharma Corporation, Chiyoda-ku, Tokyo, Japan; 3 Pharmaceuticals Research Center, Asahi Kasei Pharma Corporation, Izunokuni, Shizuoka, Japan; 4 Department of Medical Chemistry, Kansai Medical University, Hirakata, Osaka, Japan; 5 Corporate Planning and Coordination Division, Asahi Kasei Pharma Corporation, Chiyoda-ku, Tokyo, Japan; 6 Department of Rehabilitation, Osaka Kawasaki Rehabilitation University, Kaizuka, Osaka, Japan

## Abstract

Osteoporosis is a complication of diabetes mellitus (DM). The pathology of diabetic osteoporosis is distinct from postmenopausal osteoporosis, and there are no specific treatment guidelines for diabetic osteoporosis. In the current study, this issue was addressed by evaluating the effect of osteoporosis medications, such as the anabolic agent PTH [teriparatide (TPTD)] and the antiresorptive agents calcitonin [elcatonin (ECT)] and bisphosphonate [risedronate (RIS)], on bone metabolism as well as on glucose and lipid metabolism in spontaneously diabetic Torii (SDT) fatty rats, which are a model of type 2 DM (T2DM). The medicines were injected subcutaneously into 8-week-old male SDT fatty rats three times weekly for 8 weeks. TPTD treatment in SDT fatty rats increased the osteoblast number and function on trabecular bone in vertebrae, and increased the trabecular bone mass, bone mineral density (BMD), and mechanical strength of vertebrae. Additionally, TPTD improved cortical bone structure and increased BMD. RIS decreased the osteoclast number and function, which led to an increase in vertebral bone mineral content and BMD in the femoral diaphysis, and mechanical strength was increased in the vertebrae. ECT showed no clear effects on bone mass or metabolism. Similar to diabetic lesions, all of the drugs had no effects on hyperglycemia, pancreas morphology, or serum insulin and glucagon levels. However, triglyceride levels and lipid droplets in fatty liver were decreased in the TPTD group. These results suggest that TPTD may be useful for treating fatty liver in addition to osteoporosis in T2DM.

The incidence of diabetes mellitus (DM) has increased steadily in recent years. The number of adult patients will increase to 642 million by 2040, and most will have type 2 DM (T2DM) (1). T2DM is characterized by high blood glucose, insulin insensitivity as a result of insulin resistance, and impaired insulin secretion (2). Various complications have been reported in patients with T2DM, including nephropathy, retinopathy, and neurologic disorders (3). Additionally, patients with T2DM are considered to be at an increased risk of bone fracture, although bone mineral density (BMD) is normal or increased (4, 5), resulting in the development of a low-turnover osteoporosis. Recent studies revealed the deterioration of bone quality and the reduction of mechanical properties in patients with diabetes (6), which are considered to be related to impaired bone formation (7–9). Suggested causes of this reduced bone formation include decreased insulin secretion that induces bone formation via osteoblasts (10, 11) and the accumulation of bridging advanced glycation end products (AGEs) in bone collagen (12). AGEs are reported to induce apoptosis of osteoblast precursor cells and reduce osteogenic differentiation (13). Because the fracture incidence increased in association with the disease duration in patients with DM (14), therapeutic interventions that improve bone quality and mechanical properties are considered to be necessary to prevent bone fractures. The spontaneously diabetic Torii fatty [SDT.Cg-*Lepr*^*fa*^/JttJcl (SDT fatty)] rat is a novel T2DM model that was established by introducing the fa allele of the leptin receptor gene of the Zucker fatty rat into the genome of the SDT rat. The SDT fatty rats show hyperglycemia (15) and diabetic complications (16–18) from a younger age compared with the other T2DM model rats such as nonobese SDT rats (15), WBN/Kob rats, or obese WBN/Kob-*Lepr*^*fa*^ rats (19), and they are considered to be important as an animal model of nonalcoholic fatty liver disease (NAFLD) (20). Additionally, SDT fatty rats reportedly displayed lower trabecular bone volume, BMD, and mechanical strength in the femur and tibia compared with normal Sprague–Dawley (SD) rats (21). However, the effects of DM on bone turnover and microarchitecture in this T2DM rat model have not been thoroughly investigated.

Because there are no treatment guidelines that are specifically designed for diabetic osteoporosis, bisphosphonates, as antiresorptive agents, are considered to be the first-line therapy, in the same manner as for postmenopausal osteoporosis. Observational studies suggested that bisphosphonates are effective at reducing the fracture risk in patients with T2DM (22, 23), but concerns about severely suppressed bone turnover remain (24, 25).

Thus, we used the SDT fatty rat model to investigate the effects of osteoporosis drugs with different mechanisms of action, including the anabolic agent PTH [teriparatide (TPTD)] and the antiresorptive agents calcitonin [elcatonin (ECT)] and bisphosphonate [risedronate (RIS)], on bone geometry, BMD, mechanical strength, microstructure, and metabolism in trabecular and cortical bone. Additionally, the effects of these drugs on hyperglycemia and hyperlipidemia were evaluated.

## Materials and Methods

### Animal experiments

Eight-week-old male SDT fatty rats (Clea Japan, Tokyo, Japan) were obtained for the study. Only the males are commercially available, and the males develop diabetes earlier than females, with an incidence of 80% at 8 weeks and up to 100% at 16 weeks (10). Age-matched male SD Jcl:SD (SD) rats (Clea Japan) were used as control animals. The rats were maintained under a 12-hour light/12-hour dark cycle with unrestricted access to tap water and a standard diet containing 1.07% calcium, 0.83% phosphorus, 23.1 g of protein, and 137 IU of vitamin D_3_ per 100 g (MF; Oriental Yeast, Tokyo, Japan). The animals were assigned to one of the following five groups at 8 weeks of age: vehicle-treated SD rat group (SD) (n = 8); vehicle-treated SDT fatty rat group (Fatty) (n = 9); TPTD-treated SDT fatty rat group (TPTD) (n = 9); ECT-treated SDT fatty rat group (ECT) (n = 8); and RIS-treated SDT fatty rat group (RIS) (n = 8). The rats were treated with a subcutaneous injection of vehicle (SD and Fatty groups), 60 µg/kg chemically synthetized TPTD [human PTH(1–34)] (Asahi Kasei Pharma Corporation, Tokyo, Japan), 15 U/kg ECT (Asahi Kasei Pharma Corporation), or 10 µg/kg RIS (Toronto Research Chemicals, Toronto, ONT, Canada) three times per week for 8 weeks. These selected doses were shown to increase BMD in ovariectomized rats (26–28). Eight-week-old SD (n = 5) and Fatty rats (n = 5) were examined as baseline controls for the analyses of bone and lipid metabolism.

These animal studies were approved by the Ethics Committee of Wakayama Medical University and of Asahi Kasei Pharma Corporation and were conducted in accordance with guidelines concerning the management and handling of experimental animals.

### Blood sampling

Blood was collected before and 2, 4, and 8 weeks after administration. During the investigation, to avoid dietary effects on glucose and lipids, a subset of animals was fasted for 16 hours before sampling. Serum samples were obtained by centrifugation of the blood samples and were aliquoted for storage at −80°C until analysis.

### Bone sampling and preparation

Animals were euthanized under isoflurane anesthesia. The third and fifth lumbar vertebrae (L3 and L5) and the right tibias and femurs were removed. The L3 was resected at the vertebral arch and the transverse and spinous processes. A central cylinder specimen with planoparallel ends and a height of 2.7 ± 0.1 mm was obtained from each vertebral body using a diamond band saw (BS-3000; Exakt, Norderstedt, Germany). The L3 cylinders and the right femurs were stored at −20°C until measurement. The L5 and the right tibia were fixed in 70% ethanol, stained with Villanueva bone stain, dehydrated in ethanol, defatted in acetone, and embedded in poly-methyl methacrylate for bone histomorphometry.

### Bone evaluation

#### Bone geometry

The height of the lumbar vertebral cylinder and the length of the femur were determined using a digital caliper. Bone volumes were assessed using Archimedes’ principle.

#### BMD by dual energy X-ray absorptiometry

The L3 cylinders and right femurs were measured by dual X-ray absorptiometry (DCS-600EX-3R; Aloka, Tokyo, Japan). The BMD was calculated from the bone mineral content (BMC) and bone area. The femurs were divided into three equal-length regions of interest, and the middle portion was analyzed to obtain the femoral shaft BMC and BMD.

#### Mechanical strength

A compression test of the vertebral body was performed as previously described (29). Briefly, the L3 cylinder was placed on a lower plate with the cranial side facing up and compressed with an upper plate using a material testing machine (EZ-L-1kN; Shimadzu, Tokyo, Japan) at a constant speed of 2 mm/min. The load-displacement curve was recorded, and the following extrinsic parameters were calculated using the testing machine software (TRAPEZIUM2, Shimadzu): ultimate load (N), stiffness (N/mm), and breaking energy (N⋅mm).

A three-point bending test of the femoral shaft was performed as previously described (30) using the material testing machine at a constant speed of 10 mm/min. The load-displacement curves were recorded, and the following parameters were calculated: ultimate load (N), stiffness (N/mm), and breaking energy (N⋅mm).

#### Bone histomorphometry

For bone histomorphometric analysis, the animals underwent *in vivo* bone labeling by subcutaneous injection of 10 mg/kg calcein (Dojindo Laboratories, Kumamoto, Japan) at 4 and 12 days before necropsy. Bone histomorphometric parameters related to bone mass, microstructure, resorption, formation, and turnover were measured using an image analysis system (Histometry RT Camera; System Supply, Nagano, Japan).

### Trabecular bone histomorphometry

An undecalcified 5-µm-thick section was prepared from each specimen of the central L5. Histomorphometric measurements were performed with trabecular bone tissue in the secondary spongiosa region, as described in a previous study (31). The static parameters that are described below were measured for the L5, and dynamic parameters were calculated (32, 33).

#### Parameters for bone mass and structure

The following parameters were measured for bone mass and structure: trabecular bone volume (BV/TV, %), trabecular thickness (Tb.Th, µm), trabecular number (Tb.N, no./mm), and trabecular separation (Tb.Sp, µm).

#### Bone resorption parameters

The following parameters were measured for bone resorption: osteoclast number (N.Oc/BS, no./mm), osteoclast surface (Oc.S/BS, %), and eroded surface (ES/BS,%).

#### Bone formation parameters

The following parameters were measured for bone formation: osteoblast surface (Ob.S/BS, %), osteoid surface (OS/BS, %), single-labeled surface (sLS/BS, %), double-labeled surface (dLS/BS, %), interlabel thickness (Ir.L.Th, µm), and mineralizing surface (MS/BS, %).

#### Dynamic parameters

The following dynamic parameters were measured: mineral apposition rate (MAR, µm/d) and bone formation rate (BFR/BV, %/y).

### Cortical bone histomorphometry

An undecalcified 20-µm-thick cross-section was prepared from each specimen of the tibial shaft. Histomorphometric measurements were performed on periosteal and endosteal perimeters of the tibia. The static parameters that are described below were measured for the tibial cortex, and dynamic parameters were calculated (32–34).

#### Parameters of bone mass and structure

The following bone mass and structure parameters were calculated: total area (Tt.Ar, mm^2^), periosteal perimeter (Ps.Pm, mm), marrow area (Ma.Ar, mm^2^), endosteal surface (Es.Pm), cortical area (Ct.Ar, mm^2^), cortical thickness (Ct.Th, mm), cortical area (%) (Ct.Ar/Tt.Ar, %), and cortical porosity (Ct.Po, %).

#### Bone resorption parameters

The following bone resorption parameters were calculated: periosteal eroded perimeter (Ps.ES/Ps.Pm, %) and endosteal eroded perimeter (Es.ES/Es.Pm, %).

#### Bone formation parameters

The following bone formation parameters were calculated: periosteal mineralizing perimeter (Ps.MS/Ps.Pm, %) and endosteal mineralizing perimeter (Es.MS/Es.Pm, %).

#### Dynamic parameters

The following dynamic parameters were calculated: periosteal bone formation rate (Ps.BFR/Ct.Ar, %/y) and endosteal bone formation rate (Es.BFR/Ct.Ar, %/y).

### Glucose metabolism

Blood was obtained from the tail vein after 16 hours of fasting, and the glucose levels were measured using a portable blood glucose analyzer (Glutest Neo; Sanwa Kagaku Kenkyusho, Aichi, Japan). The serum insulin level was determined using an ultrasensitive rat insulin ELISA kit (M1103; Morinaga Institute of Biological Science, Kanagawa, Japan) (35), in accordance with the manufacturer’s instructions.

### Histological analysis of the pancreas and liver

Tissue samples were collected from each rat and fixed in 4% paraformaldehyde solution. The fixed tissues were dehydrated, embedded in paraffin, and 4-µm-thick sections were cut. The sections were stained with hematoxylin and eosin (H&E). As a control at the start of administration, the tissue samples of 8-week-old SD and SDT fatty rats were used. Immunohistochemical staining was performed using the avidin–biotin complex method for insulin and the EnVision method for glucagon. The primary antibodies used were anti-insulin antibody (A0564; Dako, Carpinteria, CA) (36) and anti-glucagon antibody (422271; Nichirei Biosciences, Tokyo, Japan) (37). The antigen–antibody reaction was visualized using 3,3ʹ-diaminobenzidine tetrahydrochloride as the chromogen. Slides were counterstained with Mayer’s hematoxylin. For quantitative measurement of the histological sections, digitalized images were obtained using a DM5500B microscope (Leica Microsystems, Tokyo, Japan) and a digital camera Retiga 2000R Fast1394 (QImaging, Tokyo, Japan), and five large pancreatic islets were chosen and analyzed. The areas of the pancreatic islets and immune-stained cells were determined using Image-Pro Plus (version 7.0.1.658, Media Cybernetics, Tokyo, Japan), and the insulin-producing cells mass rate was calculated as the mean insulin-positive area divided by the pancreatic islets area.

### Lipid metabolism

#### Measurement of serum and liver lipids

The liver was rapidly removed, and lipids were extracted from these tissues based on the method of Folch *et al.* (38). Briefly, snap-frozen livers were homogenized and extracted with a chloroform/methanol (2:1 v/v) solution. Total cholesterol (T-cho) and triglyceride (TG) levels in the liver were determined using commercial kits (Sekisui Medical, Tokyo, Japan). The serum T-cho and TG levels were measured using L-type Wako CHO M (Wako Pure Chemical Industries, Osaka, Japan) and L-type Wako TG M (Wako Pure Chemical Industries), respectively.

#### Liver mRNA expression related to lipid metabolism

Total RNA was extracted from the liver using the MagExtractor (NPK-201F), which was purchased from Toyobo (Osaka, Japan), after 8 weeks of drug administration. RNA was transcribed into cDNA with the iScript cDNA synthesis kit (Bio-Rad Laboratories, Berkeley, CA) using the GeneAmp PCR system 9700 (Applied Biosystems, Foster City, CA), in accordance with the manufacturer’s protocol, and real-time RT-PCR was performed using the CFX96 real-time detection system (Bio-Rad Laboratories) with iQ™ SYBR Green Supermix (Bio-Rad Laboratories). The primers were synthesized by Thermo Fisher Scientific (Waltham, MA): sterol regulatory element binding protein-1c (SREBP-1c): 5ʹ-CGACTACATCCGCTTCTTACAGC-3ʹ (forward) and 5ʹ-TTTTGTGAGCACTTCGCAGG-3ʹ (reverse); and fatty acid synthase (FAS): 5ʹ-ACTGAACGGCATTACTCGGTCC-3ʹ (forward) and 5ʹ-GTGTCCCATGTTGGATTTGGTG-3ʹ (reverse). The primers for acetyl–coenzyme A (CoA) carboxylase *α* (ACC) and *β*-actin were purchased from RealTimePrimers.com (Elkins Park, PA). The temperature profiles used for PCR of SREBP-1c and FAS comprised 45 cycles of denaturation for 15 seconds at 95°C, annealing, and extension for 60 seconds. The temperature profiles for ACC comprised 45 cycles of denaturation for 10 seconds at 95°C, annealing, and extension for 45 seconds at 58°C. The target mRNAs were normalized against the housekeeping protein *β*-actin mRNA as an internal control. The results were expressed as the ratio relative to the control. The target gene expression levels were evaluated against the *β*-actin expression level as a reference.

### Statistical analysis

All data were presented as box plots and showed median, lower, and upper quartiles, as well as minimum and maximum values. Differences between the SD and Fatty groups were assessed using Student *t* tests for all of the data. To assess the efficacy of pharmacological treatment, the TPTD, ECT, and RIS groups were compared with the Fatty group. The pharmacologically treated groups were compared with the vehicle-treated fatty rats using a Dunnett-type multiple test for all of the parameters. All statistical analyses were performed using EXSUS version 10.0 (CAC Croit, Osaka, Japan) based on SAS version 9.4 (SAS Institute Japan, Tokyo, Japan). *P* values <0.05 were considered to be statistically significant.

## Results

### Effects on bone geometry, mineral content, strength, and metabolism

#### Lumbar vertebral bodies

**Figure 1.**
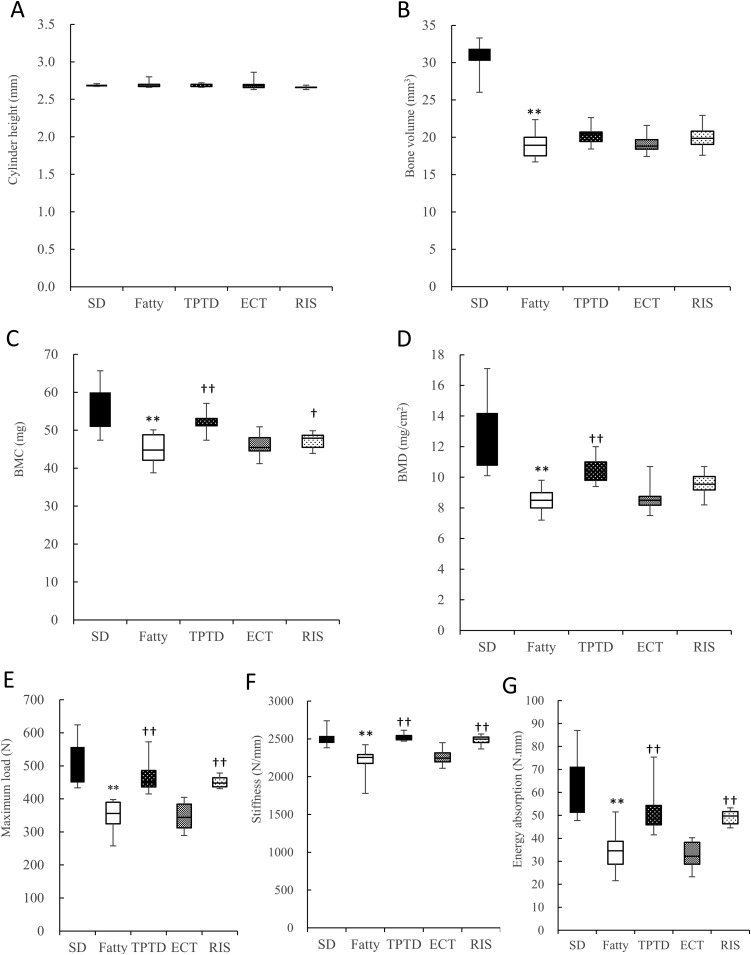
Bone geometry, mineral density, and mechanical properties of L3 bodies. Geometric parameters are (A) cylinder height and (B) bone volume. (C) BMC; (D) BMD measured by dual X-ray absorptiometry. Mechanical properties include (E) maximum load, (F) stiffness, and (G) energy absorption in a three-point bending test. All measurements were performed at the end of the administration period. The box plot shows the median, lower, and upper quartiles and the minimum and maximum values (n = 7 to 9). **P < 0.01, for difference from the SD group; ^†^*P* < 0.05, ^††^*P* < 0.01, for difference from the Fatty group.

The height of the L3 cylinders was measured to confirm that all of the specimens were trimmed to an equal-height vertebral cylinder (Fig. 1A) for the mechanical test. The bone volume of the L3 cylinder in the Fatty group was significantly lower compared with the SD group at the end of the 8-week treatment (*P* < 0.01), which was not reversed by treatment with the osteoporosis drugs (Fig. 1B). The BMC and BMD of L3 in the Fatty group were significantly lower compared with the SD group at the end of the 8-week treatment (*P* < 0.01; Fig. 1C and 1D). TPTD significantly increased the BMC and BMD of L3 compared with the Fatty group (both *P* < 0.01), whereas RIS significantly increased only the BMC (*P* < 0.05; Fig. 1C and 1D). ECT did not affect the BMC or BMD of L3 (Fig. 1C and 1D). The bone mechanical strength of L3 in the SDT fatty rats was lower compared with the SD rats (*P* < 0.01; Fig. 1E–1G). As predicted from the results of the BMC and BMD, TPTD and RIS significantly increased all of the mechanical properties (maximum load, stiffness, and energy absorption) of L3 compared with the Fatty control animals (all *P* < 0.01; Fig. 1E–1G). However, ECT did not affect the mechanical properties (Fig. 1E–1G).

#### Femurs

**Figure 2.**
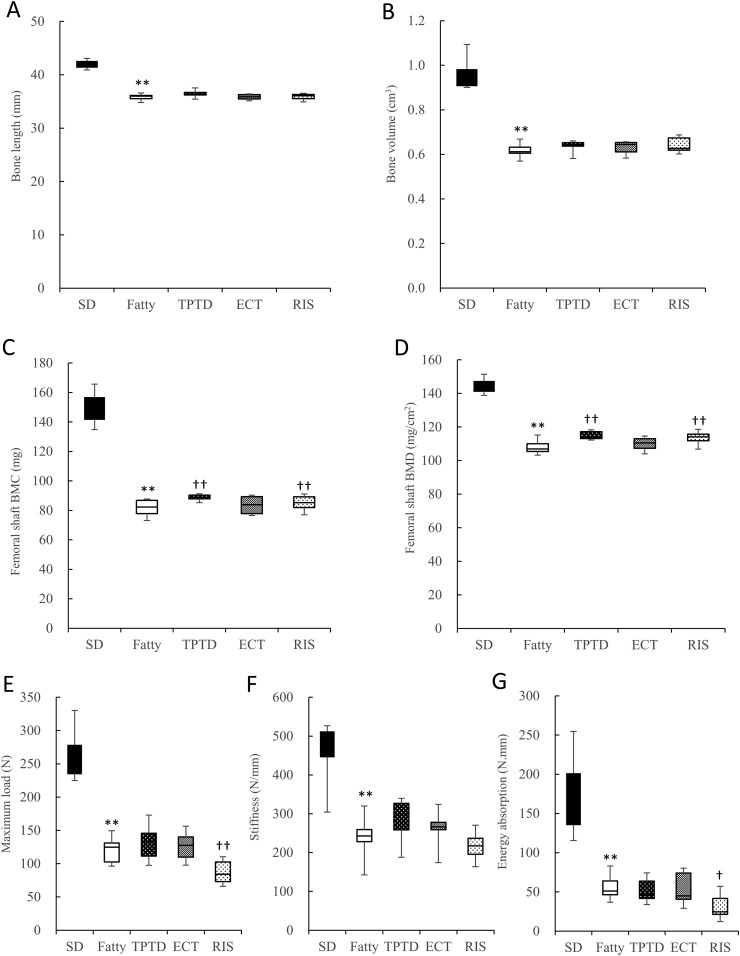
Bone geometry, mineral density, and mechanical properties of femurs. Geometric parameters include the following: (A) bone length and (B) bone volume. (C) BMC of the femoral shaft measured by dual energy X-ray absorptiometry. (D) BMD of the femoral shaft measured by dual energy X-ray absorptiometry. Mechanical properties include the following: (E) maximum load, (F) stiffness, and (G) energy absorption in a three-point bending test of the femoral diaphysis. All measurements were performed at the end of the administration period. The box plot shows the median, lower, and upper quartiles and the minimum and maximum values (n = 8 to 9). **P < 0.01, for difference from the SD group; ^†^*P* < 0.05, ^††^*P* < 0.01, for difference from the Fatty group.

The length and volume of femurs in the Fatty rats were significantly lower compared with the SD rats (*P* < 0.01; Fig. 2A and 2B). Administration of osteoporosis drugs did not affect these changes.

The BMC and BMD of the femoral shaft in the Fatty group were significantly lower compared with the SD rats (*P* < 0.01) at 16 weeks of age, which was the end of the treatment period (Fig. 2C and 2D). TPTD and RIS increased the BMC and BMD of the femoral shaft (*P* < 0.01), whereas ECT had no effect on these parameters (Fig. 2C and 2D). The bone mechanical strength in the SDT fatty rats was significantly lower compared with that in the SD rats (*P* < 0.01; Fig. 2E–2G). TPTD tended to increase the stiffness compared with the SDT fatty rats, but it was not statistically significant (*P* = 0.0857). In contrast to expectations for the BMD, RIS significantly decreased the maximum load and the energy absorption compared with the vehicle-treated SDT fatty rats (*P* < 0.01 and *P* < 0.05, respectively). ECT did not affect the femoral bone strength.

#### Bone histomorphometry

##### Trabecular bone histomorphometry of L5.

At the start of treatment when the rats were 8 weeks of age, no significant difference was observed between the SD and Fatty groups in the histomorphometric parameters, except for a reduction in the OS/BS in the Fatty group (*P* < 0.05; Table 1).

**Table 1.** Trabecular Bone Histomorphometry of L5

Parameter	Baseline		Posttreatment				
	SD (n = 5)	Fatty (n = 5)	SD (n = 8)	Fatty (n = 9)	TPTD (n = 9)	ECT (n = 8)	RIS (n = 8)
BV/TV, %	26.1 ± 3.7	27.0 ± 2.6	30.4 ± 1.7	32.4 ± 1.2	42.1 ± 1.5^*a*^	35.5 ± 1.8	37.4 ± 2.0
Tb.Th, µm	70.2 ± 5.2	72.3 ± 4.3	75.2 ± 3.6	72.4 ± 3.3	105.2 ± 4.5*^a^*	83.8 ± 4.4	82.7 ± 4.6
Tb.N, no./mm	3.67 ± 0.31	3.72 ± 0.21	4.06 ± 0.18	4.50 ± 0.08*^b^*	4.02 ± 0.09*^a^*	4.24 ± 0.09	4.54 ± 0.11
Tb.Sp, µm	211 ± 30	199 ± 15	175 ± 11	151 ± 3	144 ± 5	153 ± 6	139 ± 6
N.Oc/BS, no./mm	4.27 ± 0.40	4.33 ± 0.60	3.93 ± 0.35	4.76 ± 0.29	4.42 ± 0.46	4.46 ± 0.53	2.56 ± 0.19*^a^*
Oc.S/BS, %	12.0 ± 1.1	12.6 ± 1.9	10.3 ± 1.0	12.4 ± 0.5	13.1 ± 1.1	11.1 ± 1.2	7.0 ± 0.5*^a^*
ES/BS, %	20.4 ± 1.9	21.1 ± 2.1	13.8 ± 1.0	15.7 ± 1.0	16.3 ± 1.7	15.4 ± 1.0	9.0 ± 0.8*^a^*
Ob.S/BS, %	10.3±0.7	7.1 ± 1.7	10.8 ± 1.5	5.1 ± 0.6*^c^*	12.2 ± 1.2*^a^*	5.2 ± 0.7	6.8 ± 0.7
OS/BS, %	15.6 ± 1.1	10.3 ± 2.0*^b^*	6.70 ± 0.93	1.13 ± 0.23*^c^*	4.16 ± 0.72*^a^*	1.06 ± 0.30	0.82 ± 0.18
sLS/BS, %	18.3 ± 2.8	12.2 ± 3.2	12.7 ± 1.5	6.0 ± 1.7*^b^*	17.0 ± 3.4*^a^*	6.7 ± 1.7	14.8 ± 1.5*^d^*
dLS/BS, %*^e^*	14.2 ± 2.1	16.7 ± 3.3	8.2 ± 1.1	0.7 ± 0.3*^c^*	4.6 ± 1.1*^a^*	0.8 ± 0.4	0.2 ± 0.1
Ir.L.Th, µm*^e^*	8.66 ± 1.01	7.88 ± 0.51	9.15 ± 0.56	4.08 ± 1.40*^c^*	7.69 ± 0.70	4.78 ± 1.83	1.66 ± 0.84
MS/BS, %*^e^*	23.4 ± 2.2	22.8 ± 4.3	14.6 ± 1.4	3.7 ± 1.0*^c^*	13.1 ± 1.8*^a^*	4.2 ± 0.8	7.6 ± 0.8
MAR, µm/d*^e^*	1.44 ± 0.17	1.31 ± 0.08	1.15 ± 0.07	0.92 ± 0.13	0.96 ± 0.09	0.96 ± 0.25	0.56 ± 0.09
BFR/BV, %/y*^e^*	351 ± 43	321 ± 73	163 ± 17	43 ± 11*^c^*	88 ± 17	30 ± 10	47 ± 3

Data are expressed as the mean ± SE; n = 5 (baseline groups), n = 8 to 9 (posttreatment groups).

Abbreviations: BFR/BV, bone formation rate; BV/TV, trabecular bone volume; dLS/BS, double-labeled surface; ES/BS, eroded surface; Ir.L.Th, interlabel thickness; MAR, mineral apposition rate; MS/BS, mineralizing surface; N.Oc/BS, osteoclast number; Ob.S/BS, osteoblast surface; Oc.S/BS, osteoclast surface; OS/BS, osteoid surface; sLS/BS, single-labeled surface; Tb.N, trabecular number; Tb.Sp, trabecular separation; Tb.Th, trabecular thickness.

^a^ *P* < 0.01, for drug-treated groups vs the Fatty group (Dunnett test).

^b^ *P* < 0.05, for the Fatty group vs the SD group (unpaired Student *t* test).

^c^ *P* < 0.01, for the Fatty group vs the SD group (unpaired Student *t* test).

^d^ *P* < 0.05, for drug-treated groups vs the Fatty group (Dunnett test).

^e^ The parameters were calculated from a limited number of animals because of the disappearance of double labeling; Fatty, n = 5 of 9; ECT, n = 4 of 8; RIS, n = 3 of 8.

After the treatment period when the rats were 16 weeks old, there also was no difference between these two groups in the structural parameters (*i.e.*, the BV/TV and Tb.Th), whereas there was a significant increase in the Tb.N between the SD and Fatty groups (*P* < 0.05; Table 1). The bone-formation parameters Ob.S/BS, OS/BS, and MS/BS in the Fatty group were markedly lower compared with the SD group (*P* < 0.01; Table 1). The BFR/BV was also significantly lower in the Fatty group compared with the SD group (*P* < 0.01; Table 1).

In the SDT fatty rats, TPTD significantly increased the BV/TV and Tb.Th (*P* < 0.01), but not the Tb.N compared with the vehicle-treated SDT fatty group, whereas ECT and RIS had no effect on the bone structure parameters (Table 1). TPTD and ECT had no effect on the bone-resorption parameters, whereas RIS significantly reduced the osteoclast surface and eroded surface compared with the Fatty group (*P* < 0.01; Table 1). TPTD significantly increased the Ob.S/BS, OS/BS, and MS/BS compared with the Fatty group (*P* < 0.001), whereas ECT and RIS had no effect on the bone-formation parameters (Table 1). Although ECT and RIS did not have a significant effect on the BFR/BV compared with the Fatty group because of the disappearance of the double labeling that was required to calculate the BFR, the results in the small number of animals in which double labeling was detected suggested the suppression of bone formation in the Fatty group and the antiresorptive agents RIS and ECT groups (Table 1). However, all of the animals in the SD and TPTD groups were observed to have double labeling (Table 1), which suggests that bone formation was improved by TPTD treatment.

##### Cortical bone histomorphometry of the tibial shaft.

At baseline, when the rats were 8 weeks old, no significant difference was observed between the SD and Fatty groups in the static and dynamic parameters of cortical bone in the tibial diaphysis (Table 1). The periosteal eroded perimeter (ES/Es.Pm) was not observed in either the SD or Fatty group at the initiation of treatment (Table 2).

**Table 2.** Cortical Bone Histomorphometry of the Tibial Shaft

Parameter	Baseline		Posttreatment				
	SD (n = 5)	Fatty (n = 5)	SD (n = 8)	Fatty (n = 9)	TPTD (n = 9)	ECT (n = 8)	RIS (n = 8)
Tt.Ar, mm^2^	4.27 ± 0.47	3.93 ± 0.34	7.67 ± 0.19	4.42 ± 0.05*^a^*	4.49 ± 0.06	4.56 ± 0.09	4.55 ± 0.06
Ps.Pm, mm	12.1 ± 0.5	12.1 ± 0.3	17.2 ± 0.3	13.6 ± 0.1*^a^*	13.4 ± 0.2	13.8 ± 0.2	13.4 ± 0.2
Ma.Ar, mm^2^	1.41 ± 0.20	1.28 ± 0.13	1.80 ± 0.01	1.06 ± 0.04*^a^*	0.93 ± 0.03	1.07 ± 0.05	1.04 ± 0.05
Es.Pm, mm	6.3 ± 0.4	6.4 ± 0.4	7.4 ± 0.2	5.8 ± 0.1*^a^*	5.5 ± 0.1	5.7 ± 0.2	5.8 ± 0.1
Ct.Ar, mm^2^	2.86 ± 0.29	2.65 ± 0.21	5.87 ± 0.16	3.35 ± 0.06*^a^*	3.56 ± 0.03*^b^*	3.50 ± 0.07	3.50 ± 0.05
Ct.Th, µm	308 ± 18	286 ± 18	476 ± 11	346 ± 7*^a^*	376 ± 3*^b^*	359 ± 9	364 ± 10
Ct.Ar/Tt.Ar, %	67.3 ± 1.31	67.6 ± 0.70	76.5 ± 1.1	75.9 ± 1.0	79.4 ± 0.5*^b^*	76.7 ± 1.0	77.1 ± 0.9
Ct.Po, %	0.00 ± 0.00	0.47 ± 0.30	0.97 ± 0.36	0.75 ± 0.20	1.10 ± 0.11	0.77 ± 0.16	0.89 ± 0.23
Ps.ES/Ps.Pm, %	ND	ND	ND	ND	ND	ND	ND
Ps.MS/Ps.Pm, %	54.6 ± 5.7	49.9 ± 6.3	89.7 ± 3.2	47.5 ± 5.5*^a^*	42.5 ± 5.2	44.6 ± 6.2	52.8 ± 6.9
Ps.BFR/Ct.Ar, %/y	181 ± 74	276 ± 58	149 ± 8	62 ± 9*^a^*	45 ± 6	71 ± 12	77 ± 12
Es.ES/Es.Pm, %	ND	ND	22.9 ± 5.5	13.5 ± 3.0	13.4 ± 4.0	16.9 ± 3.7	7.0 ± 1.6
Es.OS/Es.Pm, %	45.9 ± 12.3	43.6 ± 2.8	13.6 ± 2.5	16.0 ± 2.5	21.2 ± 4.0	18.7 ± 2.3	16.6 ± 3.6
Es.MS/Es.Pm, %	66.1 ± 9.8	64.4 ± 5.7	21.5 ± 4.6	32.5 ± 2.6*^c^*	35.8 ± 3.0	33.1 ± 5.4	28.2 ± 4.0
Es.BFR/Ct.Ar, %/y*^d^*	88 ± 15	102 ± 15	13.8 ± 3.0	20.5 ± 1.6	27.5 ± 3.1	21.0 ± 3.9	22.2 ± 2.7

Data are expressed as the mean ± SE; n = 5 (baseline groups), n = 8 to 9 (posttreatment groups).

Abbreviations: Ct.Ar, cortical area; Ct.Ar/Tt.Ar, cortical area (%); Ct.Po, cortical porosity; Ct.Th, cortical thickness; Es.BFR/Ct.Ar, periosteal bone formation rate; Es.ES/Es.Pm, endosteal eroded perimeter; Es.MS/Es.Pm, endosteal mineralizing perimeter; Es.OS/Es.Pm, endosteal osteoid perimeter; Es.Pm, endosteal perimeter; Ma.Ar, marrow area; ND, not detected; Ps.BFR/Ct.Ar, periosteal bone formation rate; Ps.ES/Ps.Pm, periosteal eroded perimeter; Ps.MS/Ps.Pm, periosteal mineralizing perimeter; Ps.Pm, periosteal perimeter; Tt.Ar, total area.

^a^ *P* < 0.01, for the Fatty group vs SD group (unpaired Student *t* test).

^b^ *P* < 0.05, for drug-treated groups vs the Fatty group (Dunnett test).

^c^ *P* < 0.05, for the Fatty group vs SD group (unpaired Student *t* test).

^d^ Parameter was calculated from a limited number of animals because of the disappearance of double labeling: SD, n = 7 of 8; RIS, n = 7 of 8.

At the end of the treatment period, when the rats were 16 weeks old, the periosteal and the endosteal perimeters (Ps.Pm and Es.Pm), and cortical thickness (Ct.Th) of the tibia in the Fatty group were significantly lower compared with the SD group (*P* < 0.01), whereas there was no significant difference in the cortical bone (%) (Ct.Ar/Tt.Ar) between these two groups (Table 2). The periosteal bone formation (Ps.MS/Ps.Pm and Ps.BFR/Ct.Ar) in the Fatty group was significantly lower compared with the SD group (*P* < 0.01), whereas the endosteal bone formation (Es.MS/Es.Pm) in the Fatty group was slightly but significantly higher compared with the SD group (*P* < 0.05; Table 2). Treatment with osteoporosis drugs did not affect the length of periosteal and endosteal perimeters (Table 2). TPTD significantly increased the cortical thickness (Ct.Th) and cortical bone (%) (Ct.Ar/Tt.Ar) compared with the Fatty group (*P* < 0.05), whereas ECT and RIS did not display any significant effect on these parameters (Table 2). TPTD did not affect the endosteal eroded perimeter (Es.ES/Es.Pm), whereas the RIS group displayed a lower mean value compared with the Fatty group (48% reduction compared with the Fatty group), but this was not statistically significant because of a large variation in the Fatty control group (Table 2). There was no clear effect on the bone-formation parameters both on the periosteal and endocortical surfaces between the drug-treated groups and the vehicle-treated Fatty group (Table 2).

### Effects on glucose metabolism

**Figure 3.**
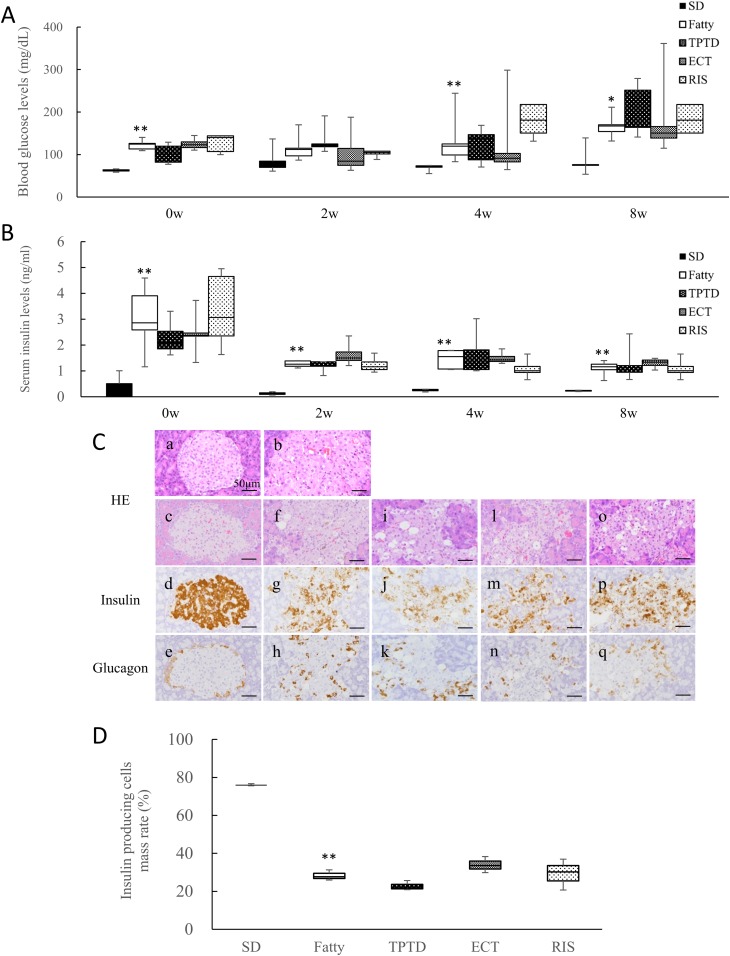
The effects of osteoporotic drugs on glucose metabolism. (A) Blood glucose levels and (B) serum insulin levels after fasting at before (0w) and after 2, 4, and 8 wk (2w, 4w, 8w) of administration (n = 3 to 5). (C) H&E staining and immunostaining for cell type–specific markers in rats with *β*-cells and *α*-cells indicated by insulin and glucagon, respectively. (a) SD rat (aged 8 wk), (b) SDT fatty rat (aged 8 wk), both before administration. (c–q) All animals were 16 wk old (*i.e.*, at the end of the administration period). (c–e) SD, (f–h) Fatty, (i–k) TPTD, (l–n) ECT, and (o–q) RIS. (D) The insulin-producing cell mass rate in pancreatic islets. Scale bars, 50 µm. The box plot shows the median, lower, and upper quartiles and the minimum and maximum values (n = 3 to 5). *P < 0.05, **P < 0.01, for difference between two groups.

The fasting blood glucose levels were significantly higher in the Fatty group compared with the SD group at 0, 4, and 8 weeks (Fig. 3A), and blood insulin levels were significantly higher at 0, 2, 4, and 8 weeks (Fig. 3B). Drug administration had no effects in the Fatty rats. For H&E staining, the pancreatic islets in the Fatty group at 8 weeks of age displayed slight vacuolation, which was increased compared with the SD group (Fig. 3C, a and b). The pancreatic islets in the Fatty group at the end of the administration (aged 16 weeks) showed hypertrophy, vacuolation, and an irregular shape compared with the SD group (Fig. 3C, c and f). In the SD group, the insulin-producing cells were located over the entire pancreatic islet, whereas the glucagon-producing cells were restricted to the margin (Fig. 3C, d and e). In the Fatty groups with or without drug treatment, the insulin-producing cells were located over the islets with a mottled appearance and there was a tendency for a low intensity of staining (Fig. 3C, g, j, m, and p). Similarly, the glucagon-producing cells of the Fatty groups were located in a scattered manner (Fig. 3C, h, k, n, and q). There were no morphological differences among these groups. Additionally, assessment of the insulin-producing cells’ mass rate was conducted by examining the mean of the insulin-positive area divided by the pancreatic islet area. TPTD treatment had the lowest insulin-producing cell mass rate among all the groups, but no significant effects with the administration of different drugs were observed (Fig. 3D).

### Effects on lipid metabolism

**Figure 4.**
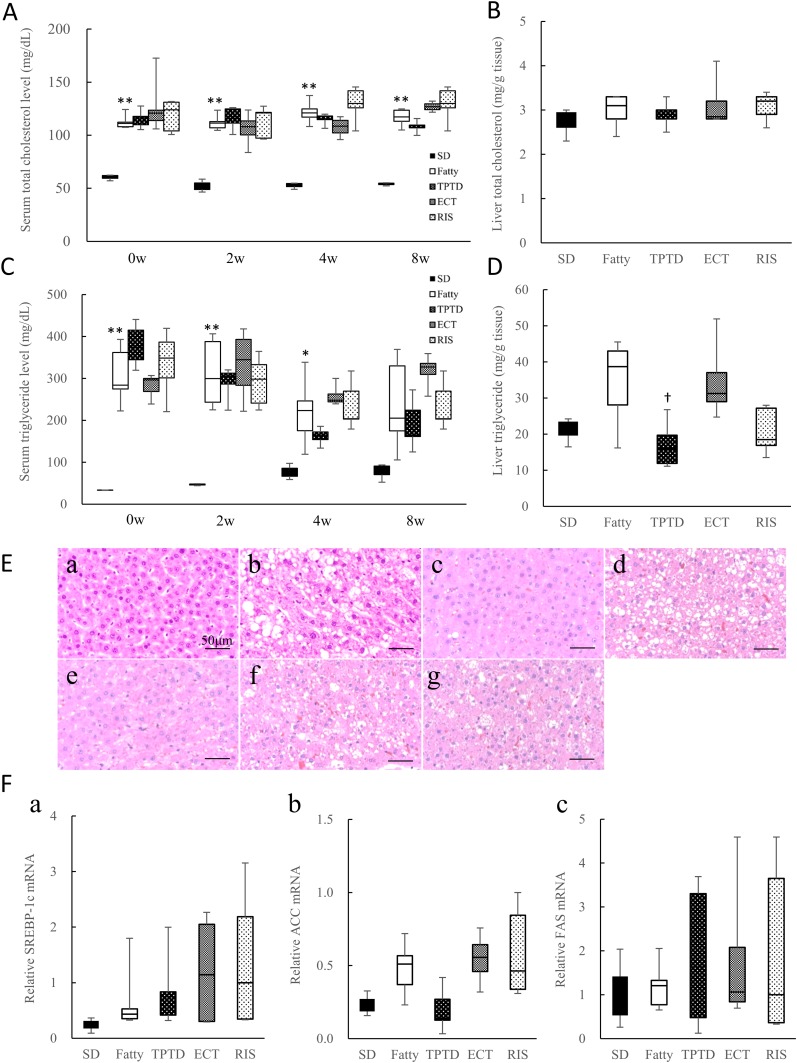
The effects of osteoporotic drugs on T-cho and TG levels in the serum and liver tissue. (A) Serum T-cho levels and (C) serum TG levels after fasting before administration (0w) and after 2, 4, and 8 wk (2w, 4w, 8w) of administration (n = 3 to 5). (B) Liver T-cho levels and (D) liver TG levels in SD rats and SDT fatty rats after 8 wk of administration (n = 3 to 5). (E) H&E staining of liver sections. (a) SD rat (8 wk old), (b) SDT fatty rat (8 wk old), both before administration. (c–g) All animals were 16 wk old (*i.e.*, at the end of the administration period). (c) SD, (d) Fatty, (e) TPTD, (f) ECT, and (g) RIS. Scale bars, 50 µm. (F) Expression of genes related to lipid metabolism. The mRNA levels were normalized to the housekeeping gene *β*-actin (n = 3 to 5). (a) Relative SREBP-1c mRNA, (b) relative ACC mRNA, and (c) relative FAS mRNA. The box plot shows the median, lower, and upper quartiles and the minimum and maximum values (n = 3 to 5). *P < 0.05, ** *P* < 0.01, for difference from the SD group; ^†^*P* < 0.05, for difference from the Fatty group.

Significant differences were observed between the SD and Fatty groups for the serum T-cho throughout the treatment period (*P* < 0.01) and for the serum TG at 0, 2, and 4 weeks (*P* < 0.05; Fig. 4A and 4C). The degree of change in the serum TG in the TPTD group was the lowest among the drug administration groups at 2, 4, and 8 weeks posttreatment (data not shown). There were no differences in the liver T-cho level between the Fatty and SD groups, and there were no effects of drug administration in the Fatty groups (Fig. 4B). The liver TG level in the TPTD group was the lowest, and it was significantly lower compared with the Fatty group (*P* < 0.05; Fig. 4D). H&E staining of the liver in the Fatty group displayed slightly more recognizable vacuolation compared with the SD group before administration (8 weeks old; Fig. 4E, a and b). This vacuolation represented lipid droplets, because the vacuoles were positively stained on Oil Red O staining (data not shown). The least amount of lipid accumulation was observed in the TPTD group among the Fatty groups treated with or without osteoporotic drugs (Fig. 4E, d–g).

Lipogenic gene (SREBP-1, FAS, ACC) expression levels in the liver were determined using real-time RT-PCR at the end of administration at 16 weeks of age. The expression of all lipogenic genes increased in the Fatty group compared with the SD group (Fig. 4F). The SREBP-1, FAS, and ACC mRNA levels in the RIS group were the highest among all five groups. The lowest SREBP-1 mRNA level was for TPTD, followed in order by ECT and RIS among the administration groups (Fig. 4F, a). TPTD showed the lowest ACC mRNA value among all of the groups, which was almost at the same level as the SD group, but it was not significantly different compared with the other drugs (Fig. 4F, b). There was no significant difference in the FAS mRNA level among the three groups treated with osteoporotic medications (Fig. 4F, c).

## Discussion

In the current study, we investigated the effects of osteoporosis drugs on bone metabolism and on glucose and lipid metabolism using the SDT fatty rat. As previously reported (21), the SDT fatty rats in the current study displayed lower bone mineral density in vertebral bodies and femurs compared with age-matched SD controls (Figs. 1 and 2). The mechanical properties of both the vertebral bodies and femoral shafts were clearly lower in the SDT fatty rats compared with SD control rats (Figs. 1 and 2). Moreover, marked suppression of bone formation, which was assessed using the osteoblast surface, osteoid surface, mineralizing surface, and bone formation rate, was revealed in the vertebral bodies of the SDT fatty rats compared with SD rats (Table 1). These results suggest that the SDT fatty rats mimic the pathology of diabetic osteoporosis in patients with T2DM, which is characterized by an increased risk of fracture and suppression of bone formation. Histopathological examination showed slight pancreatic islet vacuolation at 8 weeks of age and irregularly shaped markedly enlarged pancreatic islets with severe vacuolation after 8 weeks of treatment (16 weeks of age), and this is a characteristic finding in the SDT fatty rats. Glucose metabolism marker analyses demonstrated higher blood glucose and serum insulin concentrations in the SDT fatty rats. It is known that DM induces dyslipidemia (18). Lipid metabolism analysis showed higher serum T-cho and TG in the SDT fatty rats compared with the SD rats. NAFLD is considered to occur as fatty liver that is caused by obesity and DM, which can progress into nonalcoholic steatohepatitis as inflammation arises when part of the fatty liver deteriorates (39). Inflammatory cell infiltration and hepatic fibrosis are characteristic histological findings in nonalcoholic steatohepatitis (40). However, these were not observed in the current study, although lipid droplets in the hepatocytes were present in histological specimens of 16-week-old male SDT fatty rat livers. Thus, we consider that NAFLD was developing in these rats at this time point. It was reported that NAFLD causes a change in the BMD and it was associated with a facture risk. NAFLD was also reported to have a significant relationship with the BMD in women (41) and with the fracture risk in humans (42). Although the detailed mechanism of how NAFLD affects bone is unclear, these results strongly support our present results.

Treatment with the osteoporosis drugs TPTD and RIS showed significant effects on bone mass, bone microstructure, and mechanical properties in trabecular and cortical bones in different manners (Figs. 1 and 2). TPTD increased the BMC and BMD both in the vertebrae and femoral shaft, as previously reported by Kimura *et al.* (43) in the tibia of SDT fatty rats. TPTD also increased the mechanical properties in vertebral bodies (Fig. 1) and promoted bone formation to counter the diabetes-induced suppression (Table 1). TPTD showed a significant increase in the trabecular bone volume and trabecular thickness, which was accompanied by the increase of bone formation parameters (Table 1). Additionally, TPTD showed a significant increase in the cortical area, thickness, and cortical bone volume at the tibial shaft (Table 2), which suggests structural amelioration of the cortical bone.

RIS caused an increase in the BMC and mechanical properties in the vertebral bodies (Fig. 1), as well as a reduction in trabecular bone resorption, as assessed by the eroded surface and osteoclast surface (Table 1). Inoue *et al.* (44) reported the reduction of bone resorption markers and increased vertebral bone density by RIS treatment in osteoporosis patients with comorbid DM. These findings suggested that RIS may induce a reduction in bone resorption and turnover, which may provide an improvement in the balance between bone formation and resorption, and suppression of bone turnover would lead to a significant increase in mineralization.

No ECT-related reduction in bone resorption was demonstrated in the SDT fatty rats in the present results. Therefore, we consider that the dosing frequency, three times weekly, of ECT in this model was insufficient to yield a beneficial effect on bone metabolism.

Factors involved in the pathogenesis of diabetic osteoporosis reportedly include reduced osteoblast function because of decreased insulin secretion and bridging AGEs in bone collagen (10–12), and TPTD is a candidate that improves both of them (45).

Treatment with antiosteoporosis drugs had no clear effect on glucose metabolism in the SDT fatty rats in the current study. However, Kimura *et al.* (43) reported that 4 weeks of daily TPTD treatment (20 µg/kg/d) reduced blood glucose levels without altering the serum insulin levels in 8-week-old SDT fatty rats. This difference could be related to the number of administrations per week and the TPTD dose level. Whereas Hamann *et al.* (46) reported improved bone metabolism by PTH1–84 administration (75 µg/kg/d) in ZDF rats, no improvement in glucose metabolism was observed. These results are consistent with those of the current study, where ECT tended to have a higher ratio of insulin-positive cells in pancreatic islets and a lower blood glucose level (Fig. 3). Other researchers found significantly reduced blood glucose levels on ECT administration in male ZDF rats (47, 48), which supports our results. RIS had no clear effect on glucose metabolism in the SDT fatty rats in the current study; other researchers reported similar results in an epidemiological investigation (49).

Among the comparisons of osteoporotic drugs in the SDT fatty rats during the treatment period, TPTD showed the lowest serum TG levels. Additionally, TPTD also displayed significantly lower TG levels and fewer lipid droplets in liver tissue. It has been reported that PTH induces adipocyte lipolysis via phosphorylation of lipase (50), which strongly supports our present results, and the authors suggested that TPTD prevents NAFLD. In the future, the effects of TPTD on histological changes using older SDT fatty rats should be investigated.

Lipid synthesis–related gene expression analyses showed higher levels of SREBP-1c, ACC, and FAS gene mRNA in the SDT fatty rats compared with normal SD rats. SREBP-1c regulates FAS and ACC expression (51). ACC converts acetyl CoA to malonyl CoA, and malonyl CoA is converted to fatty acid by the action of FAS. The ACC gene expression level with TPTD was decreased compared with the SDT fatty rats and was lower than the other treatments in SDT fatty rats, but it was similar to that in the SD rats. Thus, we consider that TPTD suppresses the conversion of acetyl CoA to malonyl CoA via the inhibition of ACC gene expression through an unknown mechanism, resulting in reduced hepatic fatty acid production. In other research on fatty acid synthesis–related genes, 16-week-old female SDT fatty rats displayed disease progression and lower insulin secretion compared with normal SD rats, and these findings were accompanied by decreased SREBP-1c, FAS, and ACC expression (20). Thus, the degree of these differences was considered to vary slightly between the sexes (20).

Limitations of the current study are as follows: (i) the effect of the drugs on the advanced disease condition could not be clarified because rats of a relatively young age were used; and (ii) because the administration period was short, a longer examination period is needed in the future.

To our knowledge, this study was the first comparative investigation of the efficacy of anabolic and antiosteoporosis drugs in a T2DM animal model (SDT fatty rats). We confirmed that osteoporosis in DM results from the disruption of bone formation, and TPTD increased bone strength following the stimulation of bone formation. Additionally, TPTD decreased the TG levels and lipid droplets in fatty liver. Therefore, TPTD is considered to be potentially useful for osteoporosis and fatty liver in T2DM.

## Data Availability

The datasets generated during and/or analyzed during the current study are not publicly available but are available from the corresponding authors on reasonable request.

## Abbreviations:

ACC: acetyl–coenzyme A carboxylase *α*
AGE: advanced glycation end product
BMC: bone mineral content
BMD: bone mineral density
CoA: coenzyme A
DM: diabetes mellitus
ECT: elcatonin
ECT group: elcatonin-treated spontaneously diabetic Torii fatty rat group
FAS: fatty acid synthase
Fatty group: vehicle-treated spontaneously diabetic Torii fatty rat group
H&E: hematoxylin and eosin
L3: third lumbar vertebra
L5: fifth lumbar vertebra
NAFLD: nonalcoholic fatty liver disease
RIS: risedronate
RIS group: risedronate-treated spontaneously diabetic Torii fatty rat group
SD: Sprague–Dawley
SD group: vehicle-treated Sprague–Dawley rat group
SDT: spontaneously diabetic Torii
SDT fatty: SDT.Cg-*Lepr*^*fa*^/JttJcl
T-cho: total cholesterol
SREBP-1c: sterol regulatory element binding protein-1c
T2DM: type 2 diabetes mellitus
TG: triglyceride
TPTD: teriparatide
TPTD group: teriparatide-treated spontaneously diabetic Torii fatty rat group
